# A Novel Angle Computation and Calibration Algorithm of Bio-Inspired Sky-Light Polarization Navigation Sensor

**DOI:** 10.3390/s140917068

**Published:** 2014-09-15

**Authors:** Zhiwen Xian, Xiaoping Hu, Junxiang Lian, Lilian Zhang, Juliang Cao, Yujie Wang, Tao Ma

**Affiliations:** College of Mechantronics and Automation, National University of Defense Technology, Changsha 410073, Hunan, China; E-Mails: zwxian@nudt.edu.cn (Z.X.); jx_lian@hotmail.com (J.L.); lilian_zhang@hotmail.com (L.Z.); jlcao_nudt@163.com (J.C.); yjwang@nudt.edu.cn (Y.W.); tao.mao@nudt.edu.cn (T.M.)

**Keywords:** bio-inspired navigation sensor, polarization skylight, angle computation, sensor calibration

## Abstract

Navigation plays a vital role in our daily life. As traditional and commonly used navigation technologies, Inertial Navigation System (INS) and Global Navigation Satellite System (GNSS) can provide accurate location information, but suffer from the accumulative error of inertial sensors and cannot be used in a satellite denied environment. The remarkable navigation ability of animals shows that the pattern of the polarization sky can be used for navigation. A bio-inspired POLarization Navigation Sensor (POLNS) is constructed to detect the polarization of skylight. Contrary to the previous approach, we utilize all the outputs of POLNS to compute input polarization angle, based on Least Squares, which provides optimal angle estimation. In addition, a new sensor calibration algorithm is presented, in which the installation angle errors and sensor biases are taken into consideration. Derivation and implementation of our calibration algorithm are discussed in detail. To evaluate the performance of our algorithms, simulation and real data test are done to compare our algorithms with several exiting algorithms. Comparison results indicate that our algorithms are superior to the others and are more feasible and effective in practice.

## 1. Introduction

It is vital to know the position, velocity and attitude of an agent in many applications. Inertial Navigation System (INS) and Global Navigation Satellite System (GNSS) are conventional navigation technologies and have been widely used in many fields. INS is able to provide navigation information at a high frequency without receiving external signal, but the precision of INS decreases with time due to the accumulation of inertial sensor biases and noise [1,2]. On the contrary, GNSS can provide accurate location and velocity with bounded errors at a low frequency; however, GNSS cannot be used when the GNSS signal is unreachable or disturbed. Furthermore, the high precision INS and GNSS receivers are always bulky and expensive so they are not suitable for certain applications. What we expected is a new system, able to overcome the shortcomings of conventional navigation systems.

The remarkable navigational abilities of many animals have provided an excellent reference for us. It has been proved that the small brains of insect and animal are able to produce exquisitely efficient, robust navigation in complex environments [3,4]. The desert ants Cataglyphis, for example, are social insects (like all ants), but forage by themselves, and are able to find their way home unfailingly, in a straight line, after foraging hundreds of meters away from their nest [5]. It has been shown that Cataglyhpis get their compass direction mainly from the polarization of the blue sky [6]. Some fly insects, such as bees [7], butterfly, and dragonfly [8], are also able to utilize polarized skylight in their navigation activities. Billions of birds migrate between continents twice each year and return to the same breeding sites year after year with voyages of up to 25,000 km [9]. It is suggested that those migratory birds use skylight polarization patterns to derive an absolute directional system, which is used for calibrating all of their other compasses [10]. The polarization skylight is a natural resource of the Earth, which is caused by the scattering of sunlight in the Earth's atmosphere and is partially linearly polarized [11]. Ideas derived from the studies of insect and animal navigation behaviors have shown that the polarization of skylight can be used as a compass in navigational tasks.

**Figure 1.**
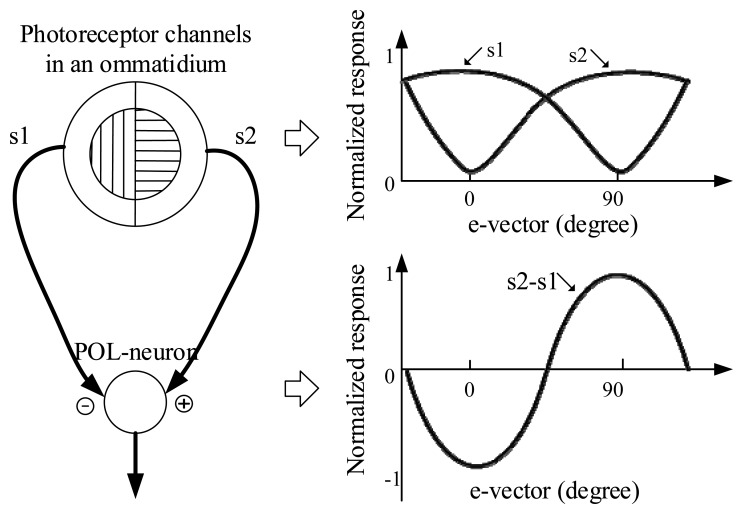
The processing of polarized-light information in the insect nervous system. The two channels in an ommatidium are tuned to orthogonal polarization directions. The e-vector response of the photoreceptor (s1,s2) have a logarithmic intensity characteristic [11]. The POL-neuron response represents a difference between s1 and s2.

According to the mechanism of processing of polarized-light in the insect, the ommatidia in the compound eyes, and the polarization-sensitive neurons (POL-neurons), are the key organs [12,13]. In each ommatidium, there are two sets of polarization-sensitive photoreceptors, which are tuned to orthogonal e-vectors (the directions of polarization). The POL-neurons receive antagonistic input from two polarization-sensitive channels with orthogonal orientations (Figure 1). This is a crossed-analyzed configuration, which enhances the polarized-light contrast sensitivity and reduces the influence of the fluctuations of light intensity. In addition, it is reported that there are three types of cricket POL-neurons which are sensitive to different e-vector directions, *i.e.*, approximately 10 deg, 60 deg, and 130 deg with respect to the body axis [14].

In order to mimic the principle of animals, researchers have done a great deal of work. Lambrinos [11,13] presented an original polarization compass model and method for extracting compass information. Chu [15–17] also constructed a POLarization Navigation Sensor (POLNS) and analyzed the sensor error with numeric simulations. A novel algorithm of the direction angle calculation was presented in [18]. In order to measure polarization information with an image array, studies on the division-of-focal-plane polarimeter have been presented in [19–21]. Furthermore, Karman [8] gave a review of bio-inspired, polarized skylight-based navigation sensors.

Studies on the POLNS mainly concentrate on the mechanical construction, the angle computation algorithm, and the sensor calibration. As for the mechanical construction of the POLNS, they have similar designs, inspired by nervous systems of insects, and ours is no exception. There are three channels, called POLarization-Opponent (POL-OP) units, in a POLNS. In terms of the angle computation algorithm, recent studies show different approaches. Lambrinos [13] and Chu [15–17] only use two of the three POL-OP units for angle computation. A transform has been utilized in [18] for angle calculation, which uses different pairs of the three POL-OP units, along with the different input polarization angles. In those angle computation algorithms, constant or selective, only two of the three POL-OP units are used, the other one is left useless, which is an unwise choice.

With regards to the sensor calibration algorithms, they have barely been reported. Zhao [22] presented an error compensation algorithms, based on LS-SVM (Least Square Support Vector Machine [23,24]). However, the main shortage of this algorithm is that it only does output angle error compensation according to the input polarization angle, which does not take the real error sources (e.g., the installation angle error and voltage bias) into consideration.

In this paper, we focus on the angle computation algorithm and the sensor calibration of a POLNS. We present a Least Squares based calculation algorithm by employing the outputs of all the POL-OP units, which can also be used for arbitrary installation angle assignment and easily expanded to multiple POL-OP unit design. As for the sensor calibration, a new approach is presented which is able to estimate the installation angles and the biases of the voltage output. Numeric simulation and real data test have been done to evaluate the performance of our angle computation and sensor calibration algorithms. Results comparison shows that, both angle computation and calibration algorithms presented in this paper have higher precision and more robustness than those of the existing algorithms.

The remainder of this paper is organized as follows: the sensor system model and the novel angle computation algorithm are detail in Section 2, followed by our calibration algorithm in Section 3. Numeric simulation and real data test are presented in Sections 4 and 5, respectively. Finally, the conclusions of this paper are given in Section 6.

## 2. Sensor Modeling and Angle Calculation

### 2.1. Sensor Overview

**Figure 2.**
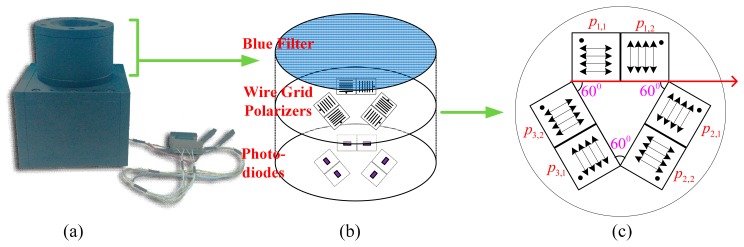
(**a**) The polarization sky-light navigation sensor, (**b**) mechanical structure, and (**c**) the ideal arrangement of three pairs of wire grid polarizers. The polarization direction of *p*_1,1_ (the red arrow in (c)) is selected as sensor reference direction.

Our sensor consists of the mechanical construction part and the circuit part. As shown in Figure 2b, there are three POL-OP units, with each POL-OP unit consisting of one pair of wire grid polarizers and one pair of photodiodes. The polarization directions of three POL-OP units are arranged in different angles. If they are ideally arranged, as shown in Figure 2c, they are adjusted by 0 degree, 60 degree, and 120 degree to the reference body axis. As for the two polarizers in a POL-OP unit, the polarization direction of one polarizer was adjusted by 90 degree to that of the other one, which mimics the crossed-analyzer configuration in the POL-area of insect eyes [13]. In reality, the actual installation angle may be different from what we want because of the mechanization processing error and the polarizer installation error. To deal with this problem, a novel angle computation algorithm is presented later in this paper.

**Figure 3.**
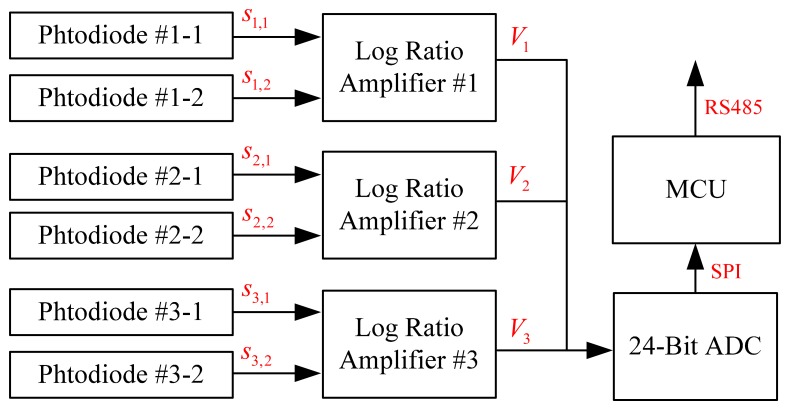
Schematic representation of the circuit signal flowchart.

Regarding the circuit of the sensor, the signal flowchart is shown in Figure 3. The photodiodes, S1087 [25], allows reliable optical measurements in the visible to near infrared range with a wide dynamic range from a low-light level to a high-light level. In order to deal with the wide range of the current coming from the photodiode, log ratio amplifiers (LOG104, [26]) are utilized, which is also a bio-inspired design [13]. Once the current is converted into voltage, a high resolution Analog Digital Converter (ADC, AD7734, [27]) is employed to transfer the analog signal to digital sequences. The digital sequences are preprocessed in an MCU (Micro Control Unit); and the data are sent to a computer through the serial communication interface of the RS485.

### 2.2. Modeling and Angle Calculation

The output current of photodiode is linear to the light level, which can be modeled as [13,18]:

(1) $$s_{i,j}\left(\phi \right)=KI(1+d\cos(2\phi -2\alpha_{i,j})),\;i=1,2,3;j=1,2$$

where *_K_* is an amplification factor related to the photodiode, *_I_* is the total intensity *I* = *I*_max_ + *I*_min_, with *I*_max_, *I*_min_ being the maximum and the minimum intensity, *_d_* denotes the polarization degree of the input light, *ϕ* is the angle between direction of input polarized light and the reference direction of the sensor, and *α_i,j_* is the maximum polarization angle of each polarizer, of which the value maximizes *s_i,j_* (*ϕ*). The output of each log ratio amplifier can be modeled as [13,18]:

(2) $$V_{i}\left(d,\phi \right)=\frac{1}{2}\log\left(\frac{1+d\cos\left(2\left(\phi -\alpha_{i,1}\right)\right)}{1+d\cos\left(2\left(\phi -\alpha_{i,2}\right)\right)}\right),i=1,2,3$$

The maximum polarization angle of each polarizer *α_i,j_* is determined by the mechanical setup, thus, it is also called installation angle. If the polarizers are ideally fixed, as shown in Figure 2c, then we have:

(3) $$\alpha_{i,j}=\frac{\pi }{3}\left(i-1\right)+\frac{\pi }{2}\left(j-1\right)\;i=1,2,3;j=1,2$$

The output of each POL-OP unit is the function of the input polarization angle *ϕ* and polarization degree *d*. The aim of the sensor is to compute those two parameters by using the outputs of POLNS outputs, which is the progress of angle computation. According to Equation (2), it is easy to know that there are two unknown parameters and three known equations. Theoretically, the two parameters can be computed by arbitrarily using two of the three equations. In reality, this idea has been used by researchers Lambrinos [13] and Zhao [18]. The difference between them is the choice of the two equations. Lamrinos used a constant two equations, while Zhao used two equations selectively according to the input polarization angle. These algorithms are feasible and can provide accurate angle estimation based on the assumption that the installation angles are known precisely and the measurement noise can be ignored. In practice, the assumption is difficult to be realized.

Contrary to the previous algorithms, a new angle computation algorithm is presented for the sake of reducing the influence of the installation angle error and measurement noise, and giving the optimal angle estimation.

We present a transform to Equation (2) as follows:

(4) $$a_{i1}d\cos\left(2\phi \right)+a_{i2}d\sin\left(2\phi \right)=y_{i},\;i=1,2,3$$

where:

(5) $$\begin{array}{l} a_{i1}=10^{2V_{i}}\cos\left(2\alpha_{i,2}\right)-\cos\left(2\alpha_{i,1}\right)\\ a_{i2}=10^{2V_{i}}\sin\left(2\alpha_{i,2}\right)-\sin\left(2\alpha_{i,1}\right)\\ y_{i}=1-10^{2V_{i}} \end{array}$$

The equation group of Equation (4) can be rewritten as:

(6) Ar=Y

where:

(7) $$\text{A}=\left[\begin{array}{cc} a_{11} & a_{12}\\ a_{21} & a_{22}\\ a_{31} & a_{32} \end{array}\right],\text{Y}=\left[\begin{array}{c} y_{1}\\ y_{2}\\ y_{3} \end{array}\right],\text{r}=\left[\begin{array}{c} r_{1}\\ r_{2} \end{array}\right]=\left[\begin{array}{c} d\cos\left(2\phi \right)\\ d\sin\left(2\phi \right) \end{array}\right]$$

In order to obtain polarization angle *ϕ* and polarization degree *d*, the vector **r** is computed first. As shown in Equations (6) and (7), there are three equations and two unknown variables. In the novel approach, we utilize the Least Squares to estimate the vector **r**. Then **r** is computed by:

(8) $$\text{r}={\left({\text{A}}^{T}\text{A}\right)}^{-1}{\text{A}}^{T}\text{Y}$$

Once **r** is obtained, the *ϕ* and *d* are computed as follows:

(9) $$\phi =\frac{1}{2}\mathrm{atan}2\left(r_{2},r_{1}\right)$$

(10) d=‖r‖

In the novel approach, a Least Squares based estimation is presented with the aim of increasing the precision and robustness of the result. In practice, all devices used in the sensor are inevitably polluted by biases and noise. With the utilization of Least Squares estimation, the residual error is minimized, thus, the optimal estimation is obtained. Moreover, this algorithm has several obvious advantages:

(1) This algorithm is more anti-noise than the existing solutions, because we use all of the POL-OP units' output and give an optimal estimation while the existing solutions adopt two of the units.
(2) The algorithm is not restricted to the fixed installation angle (e.g., 0 degree, 60 degree, and 120 degree), and can be used for an arbitrary installation angle.
(3) The algorithm is easily extended to multiple-channel design

## 3. Sensor Calibration

In regards to the sensor calibration, the error model of the sensor was analyzed first, and then the nonlinear Least Squares based calibration algorithm was deduced, and finally the implementation of the algorithm was realized.

### 3.1. Error Model

In the sensor measurement model given in Equations (1) and (2), the amplification factor *K* of each photodiode is assumed as identical; the installation angle *α_i,j_* is known precisely, and the output voltage has no bias or noise. However, it is an ideal model and not true in reality. The error model of the sensor is described as follows:

(11) $$\begin{array}{ll} {\tilde{V}}_{i}\left(d,\phi \right)=\frac{1}{2}\log\left(\frac{1+d\cos\left(2\left(\phi -{\tilde{\alpha }}_{i,1}\right)\right)}{1+d\cos\left(2\left(\phi -{\tilde{\alpha }}_{i,2}\right)\right)}\right)+n_{i}, & i=1,2,3 \end{array}$$

(12) $$\begin{array}{l} V_{i}={\tilde{V}}_{i}-b_{i},\;i=1,2,3\\ \alpha_{i,j}={\tilde{\alpha }}_{i,j}-{ɛ}_{i,j},\;i=1,2,3;j=1,2 \end{array}$$

where *Ṽ_i_*, *α̃_i,j_* are the estimated or measured values of the real values *V_i_*, *α_i.j_*, respectively, with *b_i_*, *ε_i,j_* being their error values; *n_i_* is the measurement noise of ADC, which is modeled as white noise. In this paper, *ε_i,j_* is called the installation angle error. What should be noticed is that, because we choose the axis direction of the polarizer *p*_1,1_ as the reference direction (as shown in Figure 2c), then the installation angle error related to this polarizer is zero, which is:

(13) $${ɛ}_{1,1}\equiv 0$$

As for the voltage error *b_i_*, it mainly composes of two parts, in which one is the inconsistent amplification factor *K_i,j_* and the other comes from device bias *b_device,i_*.

(14) $$b_{i}=b_{K}+b_{\text{device}}=\frac{1}{2}\log\frac{K_{i,1}}{K_{i,2}}+b_{\text{device},i}$$

The goal of our calibration algorithm is to determine installation angle errors and voltage biases.

### 3.2. Least Squares Based Calibration Algorithm

The polarization angle *ϕ* is computed by using Equations (5)–(9), which is a nonlinear function, provided that there are *N* + 1 measurements, then, *N* + 1 polarization angles can be computed as follows:

(15) $$\phi_{k}=f\left({\text{x}}_{k}\right),k=0,1,2,\ldots ,N$$

where *k* denotes the *k* th measurement, and:

(16) $${\text{x}}_{k}={\left[\alpha_{1,1},\alpha_{1,2},\alpha_{2,1},\alpha_{2,2},\alpha_{3,1},\alpha_{3,2},V_{1,k},V_{2,k},V_{3,k}\right]}^{T}$$

where *α_i,j_* can be initialized from Figure 2c and *V_i,k_* is the *k*th voltage measurement of the *i*th POL-OP unit.

In this paper, the error value, estimated value and true value of a variable or vector are defined as follows:

(17) $$a=\tilde{a}-\delta a$$

where *a* is the true value and *ã, δa* are the estimated (or measured) value and error value, respectively. As an exception, the error values of **x***_k_* and *δ***x** are slightly different, which can be obtained from Equations (12) and (13)

(18) $${\text{x}}_{k}={\tilde{\text{x}}}_{k}-\delta \text{x}$$

(19) $$\delta \text{x}={\left[0,{ɛ}_{1,2},{ɛ}_{2,1},{ɛ}_{2,2},{ɛ}_{3,1},{ɛ}_{3,2},b_{1},b_{2},b_{3}\right]}^{T}$$

It is notable that, though the last three elements of **x***_k_* are variable depending on the input, *δ***x** is constant and the first element of *δ***x** is zero because of Equation (13).

The Taylor expansion of Equation (15) is written as:

(20) $$\begin{array}{l} \phi_{k}=f\left({\tilde{\text{x}}}_{k}-\delta \text{x}\right)=f\left({\tilde{\text{x}}}_{k}\right)-\text{J}\left({\tilde{\text{x}}}_{k}\right)\delta \text{x}+Ο\left({\left\|\delta \text{x}\right\|}^{2}\right)\\ ={\tilde{\phi }}_{k}-\text{J}\left({\tilde{\text{x}}}_{k}\right)\delta \text{x}+Ο\left({\left\|\delta \text{x}\right\|}^{2}\right) \end{array}$$

where **J** is the Jacobian. This is a vector containing the first partial derivatives of the function components, which is detailed in the appendix.

Based on Equations (17) and (20), we obtain:

(21) $$\delta \phi_{k}\approx \text{J}\left({\tilde{\text{x}}}_{k}\right)\delta \text{x}$$

In the process of calibration, the relative input polarization angles are assumed as known, which is feasible in practice by using a precise rotation table. Thus, what we are able to know exactly about the input polarization light is the relative polarization angle, which is:

(22) $$\Delta \phi_{k}=\phi_{k}-\phi_{0},\;k=1,2,\ldots ,N$$

Combining Equations (20) and (22), we obtain:

(23) $$\Delta \phi_{k}={\tilde{\phi }}_{k}-{\tilde{\phi }}_{0}-\text{J}\left({\tilde{\text{x}}}_{k}\right)\delta \text{x}+\delta \phi_{0}+O\left({\left\|\delta \text{x}\right\|}^{2}\right),k=1,2,\ldots ,N$$

This equation can be rewritten as:

(24) HP=Z

where **H***_k_* ∈ **R***^N^*^×9^, **P** ∈ **R**^9^ and **Z** ∈ **R***^N^*. They are defined as follows:

(25) $$\text{H}=\left[\begin{array}{l} {\text{H}}_{1}\\ {\text{H}}_{2}\\ \ldots \\ {\text{H}}_{N} \end{array}\right],{\text{H}}_{k}=\left[{\text{J}}_{k}\left(2:9\right),-1\right]$$

(26) $$\text{P}={\left[{ɛ}_{1,2},{ɛ}_{2,1},{ɛ}_{2,2},{ɛ}_{3,1},{ɛ}_{3,2},b_{1},b_{2},b_{3},\delta \phi_{0}\right]}^{T}$$

(27) $$\text{Z}=\left[\begin{array}{l} Z_{1}\\ Z_{2}\\ \ldots \\ Z_{N} \end{array}\right],Z_{k}={\tilde{\phi }}_{k}-{\tilde{\phi }}_{0}-\Delta \phi_{k}$$

where **J***_k_*(2 : 9) ∈ **R**^8^ is the subvector of **J***_k_*, which consists of the last eight elements of **J***_k_*.

To estimate the vector **P** is our objective of sensor calibration. A least square method is fit well with this problem, and **P** can be calculated as follows:

(28) $$\text{P}={\left({\text{H}}^{T}\text{H}\right)}^{-1}{\text{H}}^{T}\text{Z}$$

Once we get an estimation of the sensor parameter **P**, we can compensate the measurement as follows:

(29) $${\text{x}}_{k}={\tilde{\text{x}}}_{k}-\delta \text{x}\left(\text{P}\right)$$

where *δ***x**(**P**) denotes that *δ***x** is the function of **P**, which can be obtained by comparing Equations (19) and (26).

To evaluate the performance of the calibration results, two indicators are given. One is to compare the estimated polarization angle with the known input polarization angle, which can be computed by using Equation (27). In other word, the vector **Z** can be used as the measurement of Least Square and also used for evaluating the results. Another variable is employed to represent the angle error:

(30) $${\text{e}}_{\phi }=\text{Z}$$

The other indicator used is to compare the measure voltage and estimated voltage as follows:

(31) $${\text{e}}_{\text{V}}={\left[e_{v1},e_{v2},\cdots ,e_{vN}\right]}^{T}$$

where:

(32) $$e_{vk}=\left\|\left[\text{V}\left({\tilde{\phi }}_{k},\tilde{\text{α}}\right)+\text{b}-{\tilde{\text{V}}}_{k}\right]\right\|$$

In this paper, the results are examined by using a linear combination of both indicators:

(33) $$e=\frac{w_{1}\left\|{\text{e}}_{\phi }\right\|+w_{2}\left\|{\text{e}}_{\text{V}}\right\|}{N}$$

where *w*_1_, *w*_2_ are weight factors, which can be arbitrarily set, but in this paper, we set that *w*_1_ = *w*_2_ = 0.5.

Furthermore, the precision of the matrix **H** depends on **x̃***_k_*. If the initial estimated **x̃** has large error, the matrix **H** also has large linearization error, and then the result computed by Equation (28) may be not accurate. In order to reduce the linearization error and improve the estimation precision, an iterative least square [28,29] implementation algorithm is employed, which is detailed in the next section.

### 3.3. Implementation of the Calibration

**Figure 4.**
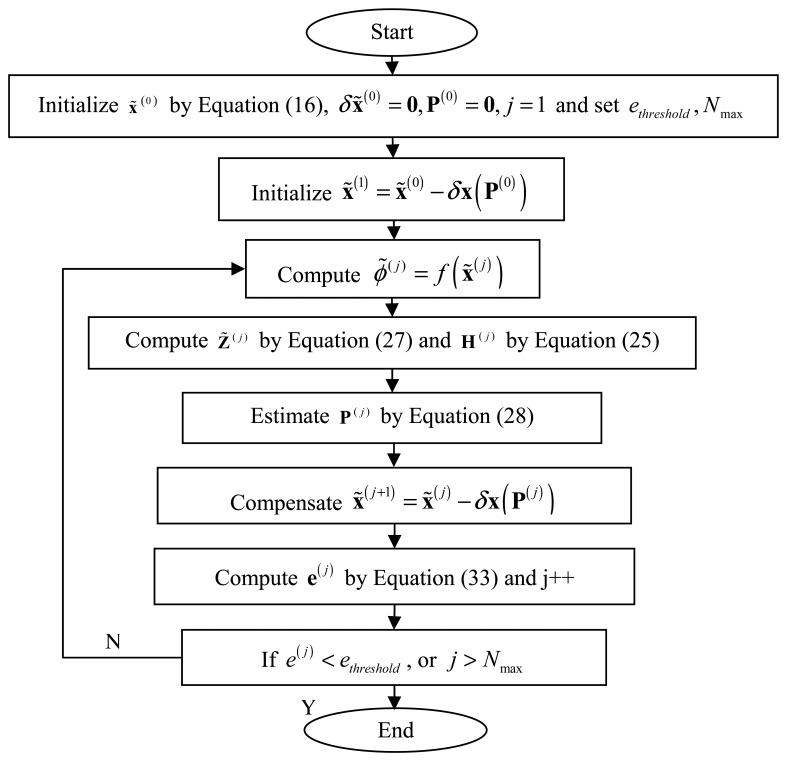
The flowchart of the implementation of our calibration algorithm.

In this section, the implementation process of iterative least square calibration is presented. What we known are the relative input polarization angle Δ*ϕ_k_* and a series of the sensor measurements $V_{i}^{k}$. The objective of calibration is to estimate the sensor parameter **P** as precisely as possible. The implementation steps are shown in Figure 4.

First, several variables **x̃**^0^, *δ***x̃**^0^, **P**^0^ are initialized and the current iterative number *j*, the indicator threshold *e_threshold_* and the maximum iterative number *N*_max_ are also set. Secondly, the initial value of **x̃**^(1)^ in obtained by compensating **x̃**^(0)^ with *δ***x̃**^0^. Then the iteration loop is started. During the iteration, the main steps contain computing *ϕ***˜**^(j)^, **Z̃**^(^*^j^*^)^,**H**^(^*^j^*^)^, estimating **P**^(^*^j^*^)^, compensating **x̃**^(^*^j^*^)^ and computing the indicator **e**^(^*^j^*^)^. After iteration, if the indicator is less than the set threshold or the current iterative number is greater than the set maximum one, the loop is stopped and the calibration results are given, otherwise, the iteration loop continues.

## 4. Simulation

In order to evaluate the performance and feasibility of our algorithm, a series of numeric simulations have been done before the test on the real data. For the angle computation algorithm, the performances of different algorithms were compared with different sensor error and noise input. Furthermore, to assess the influence of different polarization degrees, another simulation has been done. As for the calibration algorithm, a comparison was also made between the algorithm in this study and the algorithm presented by Zhao.

### 4.1. Angle Output Comparison with Different Algorithms

In order to investigate angle output performance effected by the voltage biases, the installation angle error, and the voltage noise, three different simulation are done with different error combinations, namely the voltage bias error (denoted by “B”), the voltage bias error, and installation angle error (denoted by “B+AE”), and the combination of the three types of error (denoted by “B+AE+N”). In each simulation, the results of three algorithms, namely algorithm presented by Lambranios, algorithm presented by Zhao, and our algorithm, are compared. Assuming that the polarization degree is 0.5, the voltage bias is 5 mV, the installation angle error is 1 degree, and the noise of the voltage output is white noise with the standard variance of 1 mV.

**Figure 5.**
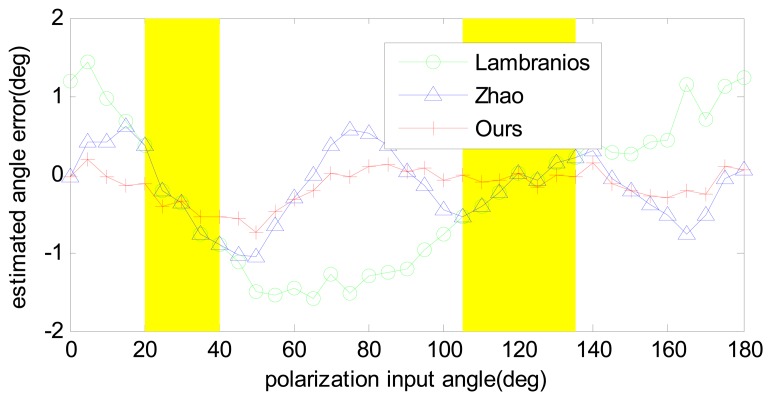
Angle estimation error comparison of different algorithms before calibration. It is clear that our algorithm shows a better performance than the other two, however, the result still has a sinusoidal trend, which indicates that a calibration is necessary in order to further improve the senor precision.

As for different error inputs, the results are shown in Table 1. As can be seen from the table, with the increase of error sources, the accuracies of all the algorithms decrease. Among the three algorithms, the result of our algorithm is much more accurate than those of the others. Particularly, the results with the input error of “B+AE+N” are compared in Figure 5. It is clear that our algorithm outperforms the other two because all of the three POL-OP units were utilized, and an optimal estimation was given based on the Least-Square. This approach is able to restrain the sensor biases and noise effectively and can be easily expanded to multiple POL-OP units. The more POL-OP units are used, the higher accuracy our algorithm will give. The results also show that, in most cases, the algorithms of Zhao are superior to those of Lambranios, however, they have the same results in certain polarization input angles (from 20 degree to 40 degree and from 105 degree to 135 degree, as shown the parts with a yellow background in Figure 5). The results can be easily explained in that Zhao chooses the optimal two POL-OP units of the three while Lambranios choose two of them constantly; when they choose the same two units they have the same results. On the other hand, when they choose different units, Zhao's algorithm shows a better performance.

**Figure 6.**
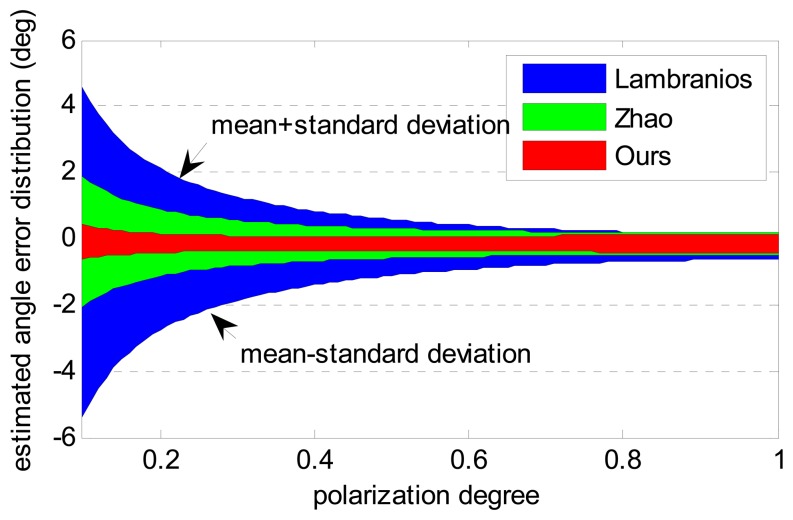
Angle estimation error comparison of different algorithm with different polarization degrees.

The three algorithms with different polarization degrees are evaluated for the sake of the study on the influence caused by polarization degree. The error source is “B+AE+N” but the polarization degree is different. As shown in Figure 6, the standard deviation of the estimated angle error decreases as the polarization degree increases. The reason is that the amplitude of voltage output has a positive correlation with the polarization degree. For the same error source, the signal to noise ratio also increases as the voltage amplitude increases. Therefore, all the algorithms show a better performance in high polarization degrees. Our algorithm, however, has similar precision, even in low polarization degrees. The simulation results show that our algorithm is more accurate and robust than the others.

### 4.2. Calibration Results

In order to test the performance of the iterative Least Squares based calibration algorithm, the algorithm is tested with large initial errors, which is very important in a real calibration process. Taking the installation angle calibration for example, before calibration, an initial value of the angle with bounded error should be given. If our algorithm is able to be convergent for a large initial error, then just an inexact value of the installation angle is needed by using a common protractor. As shown in Table 2, there are eight calibration parameters, the initial installation angle error is four degrees, and the voltage bias are set to 10 mV. Before calibration, the parameters are all assumed as zeros. After the first calibration loop, an estimation of the parameters is obtained which is quite unacceptable, however, an accurate estimation is obtained after five iterations, with the installation angle error being about 0.01 deg, and the voltage bias being about 0.03 mV. The simulation results show the accuracy and robustness of our calibration algorithm.

**Figure 7.**
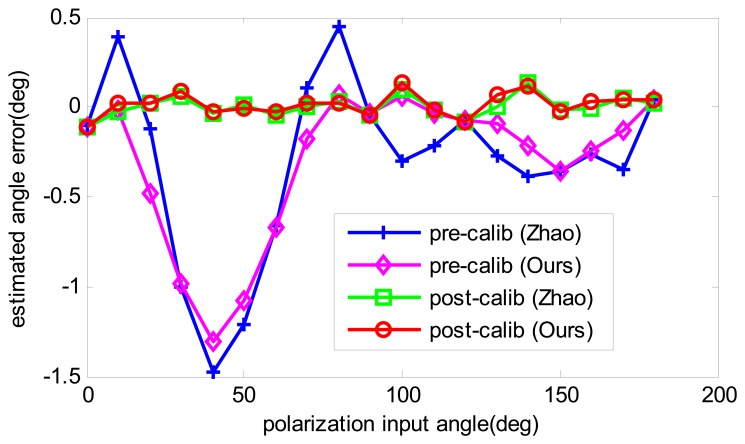
Estimated angle error comparison of pre-calibration and post-calibration.

**Figure 8.**
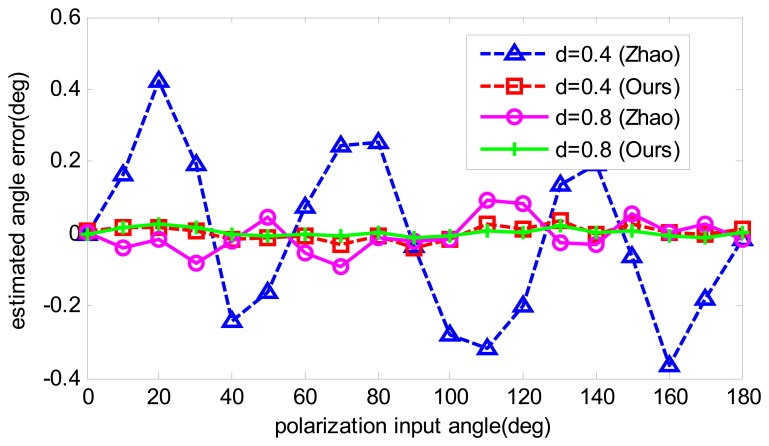
Estimated angle error comparison of post-calibration with different polarization degrees.

To compare the angle estimation precision before calibration and after calibration, the angle estimation with calibrated parameters is recalculated. Firstly, the data that are also used for calibration are applied to compare the calibration performance. The results are shown in Figure 7. Both calibration algorithms have improved the angle computation precision, and have a similar accuracy after calibration. However, when another two groups of numeric data are utilized, which have the same error sources but different polarization degrees, the results of the two calibration methods show differently. As shown in Figure 8, it is obvious that our algorithm has much more precision than Zhao's algorithm at the polarization degrees of 0.4 and 0.8, which enables our algorithm to be more applicable in practice. The reason for this is that Zhao's algorithm only performs curve-fitting for the angle error and compensates for it, which may be useful if the sensor has the same error trait. However, when the error trait changes, the accuracy decreases. On the contrary, in our algorithm, the aim is to find the real error resource and compensate for it, which is independent of the polarization degree and polarization input angle. Thus, our calibration algorithm is superior to Zhao's algorithm.

## 5. Real Data Test

### 5.1. Experiment Setup

**Figure 9.**
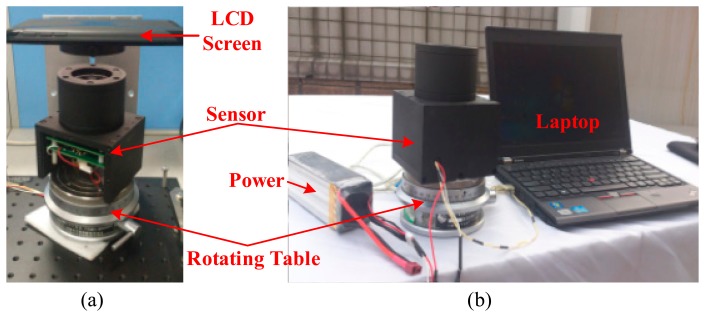
Polarization navigation sensor test setup. (**a**) Sensor calibration test (**b**) Outdoor test.

To verify the performance of the novel algorithm of angle computation and sensor calibration in a real system, the algorithms are tested with our bio-inspired polarization navigation sensor. The test system consists of a 24 V Li-Po battery, a precise angle-dividing table, which is able to provide accurate rotating angle discretely with the minimal step of 360/391 degree, a polarization sensor, and a laptop for saving the sensor voltage output. The angle-dividing table rotates horizontally driven by hand with the precision of 0.0001 degree at each step, which is accurate enough for the test. As for the calibration test, to get a standard polarization light source, an integrating sphere is used in [17], however, due to the lack of a sphere, an LCD screen of a tablet PC is utilized to provide the standard polarization light, as shown in Figure 9a. Furthermore, an outdoor test (as shown in Figure 9b) is also done to evaluate the performance of our calibration algorithm.

### 5.2. Results

**Figure 10.**
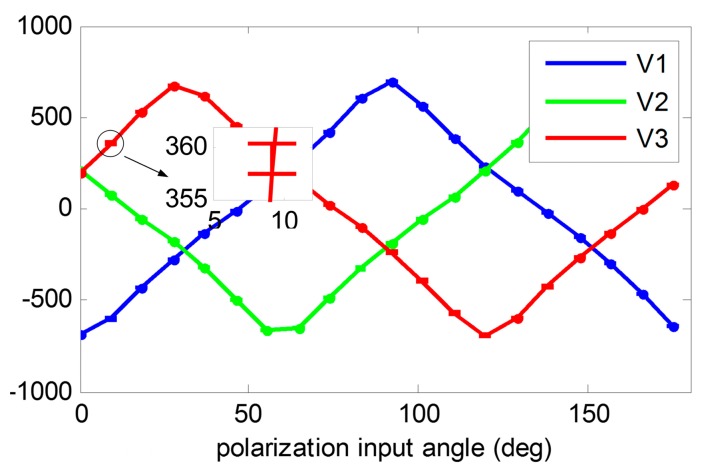
Raw voltage outputs at the 20 steps during the sensor calibration test. Each step contains about 1000 epochs. The data of voltage 3 at Step 2 have been amplified, in which the two bars denote the minimum value and maximum value of the sample data. The voltage output is stable, as shown in the amplificatory figure, with the noise of the voltage measurement being less than 5 mV.

For calibration, the angle-dividing table is rotated every 10 scales for each step and the table is kept static for about 10 s. The data are recorded at a sample rate of 100 Hz. There are a total of 20 steps with each step being 9.2072 degree. The raw voltage outputs are shown in Figure 10. After recording the data, 10 epochs of each step are randomly selected for calibration. The initial installation angles are obtained from the mechanical CAD design, which are also with the ideal values. The estimated calibration parameters are shown in Table 3. The result shows that the installation angle errors ranged from 0 to 3 degree and the voltage biases are nearly 10 mV. These errors are relatively large so it is very necessary to do the calibration before using the POLNS.

**Figure 11.**
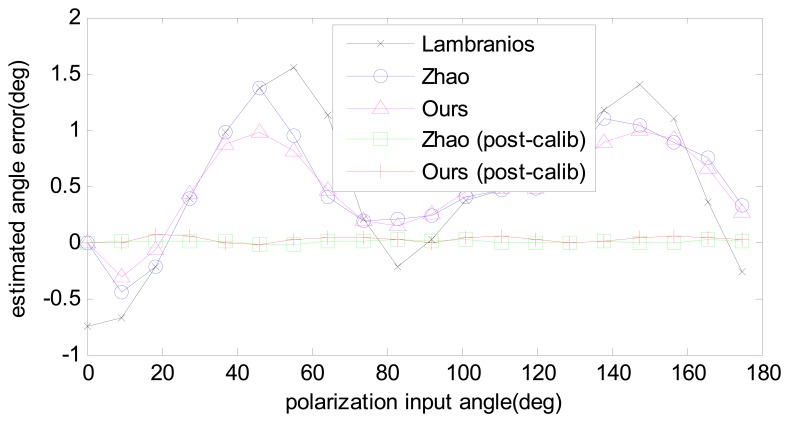
Angle computation results with real data. Three angle computation algorithms, namely Lambranios, Zhao, and ours, are tested before calibration. The results of post-calibration of Zhao's algorithm and our calibration algorithm are also shown in the figure.

The same data are processed with three different angle computation algorithms and two calibration algorithms. The angle computation errors are compared in Figure 11. It is clear to see that the error trait of three angle computation algorithms have the similar trends. The reason is that the input light source has relative high polarization degree, to be exact; it is 0.9 for this calibration. According to the simulation results shown in Figure 6, the three algorithms have fewer differences in the high polarization degree. Even so, our algorithm shows a better performance than the other two. After calibration and error compensation, the results of Zhao's algorithm was more or less the same as ours.

**Figure 12.**
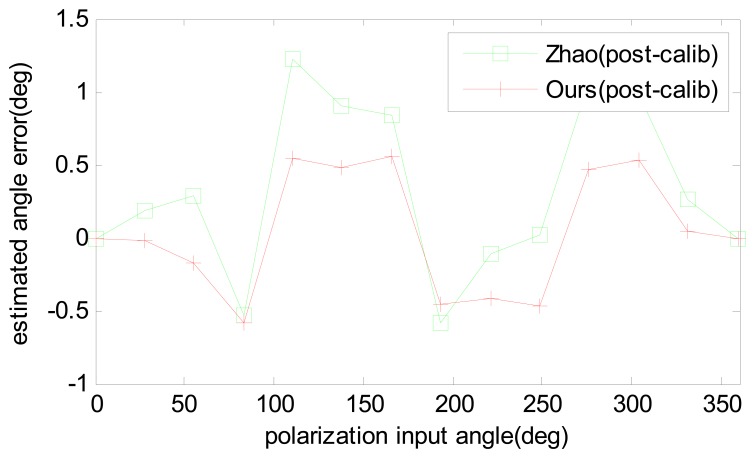
Estimated angle error comparison of the post-calibration with outdoor test data.

**Figure 13.**
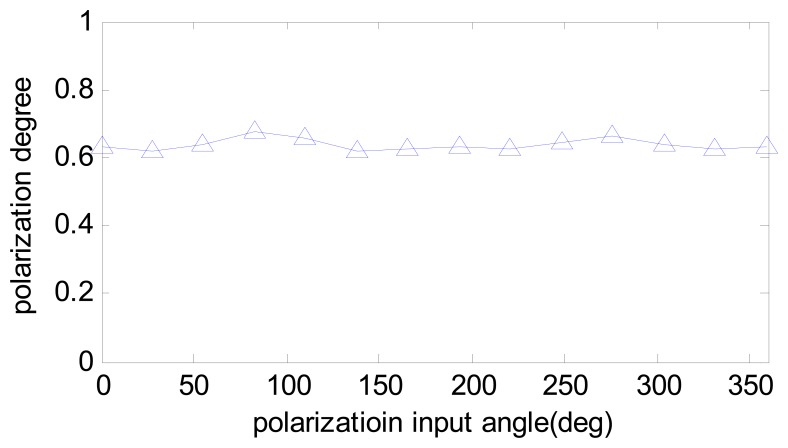
The computed polarization degree of outdoor test.

In reality, the polarization degree of skylight is barely able to reach 0.9. The calibrated parameters are used to compensate one experiment done in the outdoor. The results are compared in Figure 12 and the computed polarization degree is shown in Figure 13. The maximum angle error is about 0.5 degree, which is half of the Zhao's algorithm. The result shows that our calibration algorithm is more robust and useful than Zhao's. Furthermore, it can be noticed that both algorithms have a relative large error of numeric simulation, which is because of unstable sky polarization pattern, which may be caused by moving clouds and the movement of the sun during the real data test.

## 6. Conclusions

In the work presented here, we have studied two important issues of bio-inspired POLNS: input polarization angle computation and sensor calibration. Regarding the angle computation algorithm, conventional solutions use only two units of the three POL-OP units of the POLNS, in which sensor output information is not fully utilized. Based on Least Squares algorithm and our sensor model, a novel angle computation algorithm is derived, which is more anti-noise than the existing ones, and can be used for an arbitrary mechanical installation angle and is easily extended to multi-unit designs. With regards to sensor calibration, the main error sources of POLNS, installation angle errors and voltage biases, were analyzed first. Then, the calibration algorithm, based on a standard polarization light source and a precise turntable, was deduced. The aim of the calibration algorithm is to estimate the original sensor parameters and biases, which enables the algorithm to perform better than exiting algorithm.

The proposed two algorithms have been applied to several numeric simulations and real data tests. The comparison with existing algorithms shows that our angle computation algorithm has the same level of precision for different polarization degree input, is more accurate and robust than the other algorithms for the same error input, and that our calibration algorithm is more effective and useful in practice. With the results seen herein, simulation and actual test results are consistent with the analysis, which shows the feasibility and superiority of our algorithms.

## Figures and Tables

**Table 1.** Angle estimation error of three algorithms with different error inputs.

**Algorithm**	**Lambranios**			**Zhao**			**Ours**		
Err Source	B	B+AE	B+AE+N	B	B+AE	B+AE+N	B	B+AE	B+AE+N
Max Err(deg)	1.07	1.44	1.60	0.64	1.11	1.06	0.15	0.63	0.75
Avg Err(deg)	0.03	−0.21	−0.22	−0.00	−0.16	−0.15	0.00	−0.16	−0.15
Std Err(deg)	0.84	0.94	0.94	0.40	0.44	0.46	0.10	0.20	0.22

**Table 2.** Parameters estimation of iterative Least Square based calibration algorithm.

**Params**	*_ε_*_1,2_	*_ε_*_2,1_	*_ε_*_2,2_	*_ε_*_3,1_	*_ε_*_3,2_	*_b_*_1_	*_b_*_2_	*_b_*_3_
units	(deg)	(deg)	(deg)	(deg)	(deg)	(mV)	(mV)	(mV)
True value	4.00	−4.00	4.00	4.00	−4.00	−10.00	10.00	−10.00
IterNum = 0	0.00	0.00	0.00	0.00	0.00	0.00	0.00	0.00
IterNum = 1	4.77	−3.02	3.51	4.53	−3.82	−9.70	9.88	−8.57
IterNum = 2	4.00	−4.00	4.02	4.02	−4.01	−9.93	10.04	−9.96
IterNum = 5	4.00	−4.01	4.00	4.00	−4.01	−9.97	10.03	−10.00

**Table 3.** Calibration parameter estimation of our real system.

**Parameters**	*_α_*_1,1_	*_α_*_1,2_	*_α_*_2,1_	*_α_*_2,2_	*_α_*_3,1_	*_α_*_3,2_	*_b_*_1_	*_b_*_2_	*_b_*_3_
units	(deg)	(deg)	(deg)	(deg)	(deg)	(deg)	(mV)	(mV)	(mV)
initial	0.00	60.0	120.0	90.0	150.0	210.0	0	0	0
final	0.00	58.6	117.9	88.6	147.4	209.0	−1.2	−8.9	−5.4

## Appendix: Calculation of the Jacobian

This section, shows the process of calculating the Jacobian **J**(**x***_k_*), which are the partial derivatives of the nonlinear Equation (15). For simplify, **J** is utilized to denote the Jacobian in short in the following.

(A.1) $$\text{J}\left(\text{x}\right)=\left[\frac{\partial f}{\partial \alpha_{1,1}},\frac{\partial f}{\partial \alpha_{1,2}},\frac{\partial f}{\partial \alpha_{2,1}},\frac{\partial f}{\partial \alpha_{2,2}},\frac{\partial f}{\partial \alpha_{3,1}},\frac{\partial f}{\partial \alpha_{3,2}},\frac{\partial f}{\partial V_{1}},\frac{\partial f}{\partial V_{2}},\frac{\partial f}{\partial V_{3}}\right]$$

In order to deduce the partial derivatives, some intermediate variables are introduced as follows:

(A.2) $$\text{D}=\left[\begin{array}{cc} D_{1} & D_{3}\\ D_{3} & D_{2} \end{array}\right]=\left({\text{A}}^{T}\text{A}\right)=\left[\begin{array}{cc} \sum_{i=1}^{3}a_{i1}^{2} & \sum_{i=1}^{3}a_{i1}a_{i2}\\ \sum_{i=1}^{3}a_{i1}a_{i2} & \sum_{i=1}^{3}a_{i2}^{2} \end{array}\right]$$

(A.3) $$\text{B}=\left[\begin{array}{l} B_{1}\\ B_{2} \end{array}\right]=\text{A}Y=\left[\begin{array}{l} \sum_{i=1}^{3}a_{i1}y_{i}\\ \sum_{i=1}^{3}a_{i2}y_{i} \end{array}\right]$$

Then the vector **r** can be computed by:

(A.4) $$\text{r}=\frac{1}{T_{3}}\left[\begin{array}{l} T_{1}\\ T_{2} \end{array}\right]={\text{D}}^{-1}\text{B}=\frac{1}{D_{1}D_{2}-D_{3}^{2}}\left[\begin{array}{c} D_{2}B_{1}-D_{3}B_{2}\\ -D_{3}B_{1}+D_{1}B_{2} \end{array}\right]$$

According to Equation (9), we get the derivatives of *ϕ* with respect to **r**

(A.5) $${\text{J}}_{\phi }\left(\text{r}\right)=\frac{1}{2\left\|\text{r}\right\|}\left[-r_{2},r_{1}\right]$$

The following derivatives can also be deduced as:

(A.6) $${\text{J}}_{\text{r}}\left(\text{T}\right)=\frac{1}{T_{3}^{2}}\left[\begin{array}{ccc} T_{3} & 0 & -T_{1}\\ 0 & T_{3} & -T_{2} \end{array}\right]$$

Since the variable **T** is the function of **D** and **B**, as shown in Equation (A.4)**D** and **B** are substituted into one variable **G** = [**D***^T^*, **B***^T^*]*^T^* = [*D*_1_, *D*_2_, *D*_3_, *B*_1_, *B*_2_]^*T*^. Then, we can obtain:

(A.7) $${\text{J}}_{\text{T}}\left(\text{G}\right)=\left[\begin{array}{ccccc} 0 & B_{1} & -B_{2} & D_{2} & -D_{3}\\ B_{2} & 0 & -B_{1} & -D_{3} & D_{1}\\ D_{2} & D_{1} & -2D_{3} & 0 & 0 \end{array}\right]$$

Additionally, **A** and **Y** are substituted into one vector:

(A.8) $$\text{L}={\left[{\text{A}}^{T}(:,1),{\text{A}}^{T}(:,2),{\text{Y}}^{T}\right]}^{T}$$

where **A** (:,1), **A**(:,2) are the first column vector and the second column vector of **A**, respectively. Then, we have:

(A.9) $${\text{J}}_{\text{G}}\left(\text{L}\right)=\left[\begin{array}{ccc} 2{\text{A}}^{T}\left(:,1\right) & {\text{0}}_{1\times 3} & {\text{0}}_{1\times 3}\\ {\text{0}}_{1\times 3} & 2{\text{A}}^{T}\left(:,2\right) & {\text{0}}_{1\times 3}\\ {\text{A}}^{T}\left(:,2\right) & {\text{A}}^{T}\left(:,1\right) & {\text{0}}_{1\times 3}\\ {\text{Y}}^{T} & {\text{0}}_{1\times 3} & {\text{A}}^{T}\left(:,1\right)\\ {\text{0}}_{1\times 3} & {\text{Y}}^{T} & {\text{A}}^{T}\left(:,2\right) \end{array}\right]$$

As for the last derivative **J_L_**(**x**), it is computed as follows:

(A.10) $${\text{J}}_{\text{L}}\left(\text{x}\right)=\left[\begin{array}{ccc} {\text{J}}_{11} & {\text{0}}_{1\times 3} & {\text{0}}_{1\times 3}\\ {\text{0}}_{1\times 3} & {\text{J}}_{22} & {\text{0}}_{1\times 3}\\ {\text{0}}_{1\times 3} & {\text{0}}_{1\times 3} & {\text{J}}_{33}\\ {\text{J}}_{41} & {\text{0}}_{1\times 3} & {\text{0}}_{1\times 3}\\ {\text{0}}_{1\times 3} & {\text{J}}_{52} & {\text{0}}_{1\times 3}\\ {\text{0}}_{1\times 3} & {\text{0}}_{1\times 3} & {\text{J}}_{63}\\ {\text{J}}_{71} & {\text{0}}_{1\times 3} & {\text{0}}_{1\times 3}\\ {\text{0}}_{1\times 3} & {\text{J}}_{82} & {\text{0}}_{1\times 3}\\ {\text{0}}_{1\times 3} & {\text{0}}_{1\times 3} & {\text{J}}_{93} \end{array}\right]$$

where:

(A.11) $$\begin{array}{l} {\text{J}}_{11}=\left[\begin{array}{ccc} 2\sin\left(2\alpha_{1,1}\right) & -2\times 10^{2V_{1}}\sin\left(2\alpha_{1,2}\right) & 2\log(10)\times 10^{2V_{1}}\cos\left(2\alpha_{1,2}\right) \end{array}\right]\\ {\text{J}}_{22}=\left[\begin{array}{ccc} 2\sin\left(2\alpha_{2,1}\right) & -2\times 10^{2V_{2}}\sin\left(2\alpha_{2,2}\right) & 2\log(10)\times 10^{2V_{2}}\cos\left(2\alpha_{2,2}\right) \end{array}\right]\\ {\text{J}}_{33}=\left[\begin{array}{ccc} 2\sin\left(2\alpha_{3,1}\right) & -2\times 10^{2V_{3}}\sin\left(2\alpha_{3,2}\right) & 2\log(10)\times 10^{2V_{3}}\cos\left(2\alpha_{3,2}\right) \end{array}\right]\\ {\text{J}}_{41}=\left[\begin{array}{ccc} -2\cos\left(2\alpha_{1,1}\right) & 2\times 10^{2V_{1}}\cos\left(2\alpha_{1,2}\right) & 2\log(10)\times 10^{2V_{1}}\sin\left(2\alpha_{1,2}\right) \end{array}\right]\\ {\text{J}}_{52}=\left[\begin{array}{ccc} -2\cos\left(2\alpha_{2,1}\right) & 2\times 10^{2V_{2}}\cos\left(2\alpha_{2,2}\right) & 2\log(10)\times 10^{2V_{2}}\sin\left(2\alpha_{2,2}\right) \end{array}\right]\\ {\text{J}}_{63}=\left[\begin{array}{ccc} -2\cos\left(2\alpha_{3,1}\right) & 2\times 10^{2V_{3}}\cos\left(2\alpha_{3,2}\right) & 2\log(10)\times 10^{2V_{3}}\sin\left(2\alpha_{3,2}\right) \end{array}\right]\\ {\text{J}}_{71}=\left[\begin{array}{ccc} 0 & 0 & -2\log(10)\times 10^{2V_{1}} \end{array}\right]\\ {\text{J}}_{82}=\left[\begin{array}{ccc} 0 & 0 & -2\log(10)\times 10^{2V_{2}} \end{array}\right]\\ {\text{J}}_{93}=\left[\begin{array}{ccc} 0 & 0 & -2\log(10)\times 10^{2V_{3}} \end{array}\right] \end{array}$$

Once all the intermediate derivatives have been calculated, the Jacobian is obtained:

(A.12) $$\text{J}\left(\text{x}\right)={\text{J}}_{\phi }\left(\text{r}\right){\text{J}}_{\text{r}}\left(\text{T}\right){\text{J}}_{\text{T}}\left(\text{G}\right){\text{J}}_{\text{G}}\left(\text{L}\right){\text{J}}_{\text{L}}\left(\text{x}\right)$$
